# Effect of oxyresveratrol under in vitro lipopolysaccharide-induced periodontitis environment

**DOI:** 10.1186/s12903-024-05128-2

**Published:** 2024-11-15

**Authors:** Ju Ri Ye, Seung Hwan Park, Sang Wook Kang, Kyu Hwan Kwack, Yong Kwon Chae, Hyo-Seol Lee, Sung Chul Choi, Ok Hyung Nam

**Affiliations:** 1 Department of Dentistry, Graduate School, Kyung Hee University, Seoul, Korea; 2 Department of Pediatric Dentistry, Kyung Hee University College of Dentistry, Kyung Hee University Medical Center, Seoul, Korea; 3 Department of Oral and Maxillofacial Pathology, School of Dentistry, Kyung Hee University, Seoul, Korea; 4 Department of Oral Microbiology, College of Dentistry, Kyung Hee University, Seoul, Korea; 5 Department of Pediatric Dentistry, School of Dentistry, Kyung Hee University, Kyungheedae-Ro 26, Dongdaemoon-Gu, Seoul, 02447 Korea

**Keywords:** Cytokines, Inflammation, Natural product, Periodontal ligament cell, Periodontitis

## Abstract

**Background:**

Oxyresveratrol is the main constituent of mulberries and has many bioactive properties beneficial to human health. The purpose of this study was to assess the anti-inflammatory effects of oxyresveratrol on in vitro periodontitis model.

**Methods:**

Human periodontal ligament cells were treated with oxyresveratrol (0, 10, and 20 µg/mL) for 72 h. Cell viability and flow cytometry assays were performed. To investigate anti-inflammatory effect of oxyresveratrol on periodontal inflammation, nitric oxide production under lipopolysaccharide stimulation was assessed. Next, expression of biomarkers associated periodontal inflammation was evaluated. Scratch wound assay was performed to evaluate cell migration/proliferation potential of oxyresveratrol under lipopolysaccharide stimulation.

**Results:**

Periodontal ligament cell toxicity was not observed in oxyresveratrol treatment. Oxyresveratrol treatment significantly inhibited nitric oxide production and reduced MMP-2, MMP-9, TNF-α, IL-6, and IL-8 expressions after lipopolysaccharide stimulation. Regarding cell migration/proliferation, open wound area in oxyresveratrol (33.28 ± 6.80%) was the lowest (*p* < 0.05).

**Conclusions:**

Within the limits of this study, oxyresveratrol inhibited lipopolysaccharide-induced inflammation in periodontal ligament cells and promoted periodontal ligament cell migration/proliferation. These findings suggest that oxyresveratrol could be valuable for the management of periodontal diseases.

**Supplementary Information:**

The online version contains supplementary material available at 10.1186/s12903-024-05128-2.

## Background

Periodontitis is characterized by the breakdown of underlying periodontal tissue [1]. Periodontal disease, which causes serious problems in the oral cavity, affects 20‒50% of the adult population in the world [2]. Once periodontitis begins, inflammation causes deep periodontal pockets to form, which in turn creates an environment for bacteria and microorganisms to grow, leading to disease progression [3]. Among the microbial pathogens, *Porphyromonas gingivalis* is highly associated with the pathogenesis of periodontitis [4]. According to the literature, lipopolysaccharide (LPS) produced by *Porphyromonas gingivalis* is mainly responsible for the periodontal tissue destruction [5]. With periodontitis progression, LPS modulates periodontal inflammation by releasing inflammation-related cytokines [6–8].

Ideally, treatment for periodontitis will involve periodontal tissue regeneration [9]. Periodontal ligmanent (PDL) cells are key players for periodontal regeneration, because they can turn into a wide range of progenitor cells, including periodontal fibroblasts, cementoblasts and osteoblasts [10]. However, the current treatment strategies for periodontitis are limited to subgingival infection control and mechanical removal of periodontal pockets [11]. Because periodontal regeneration is hardly achieved by the current treatment strategies, bone grafts have been used to promote the regeneration [9]. Along with the strategies, a combination of an antibiotic agent and mechanical therapy is also being implemented [12]. However, side effects and resistance of antibiotics agents may remain to be concerned [13]. Collectively, there may be a need for the finding of natural compounds for periodontal regeneration [14, 15].

Oxyresveratrol (OXY) is a polyhydroxystilbene found in mulberries [16]. Mulberries are highly nutritious and offer health benefits for human diseases, including anti-diabetic, anti-hypertensive, and anti-obesity properties [17]. Ingredients in mulberries, such as mulberroside A, resveratrol, and OXY possess anti-oxidant activity [18]. Among the ingredients, OXY is known to be the main contributor for anti-oxidant activity [19]. In addition, previous studies found anti-inflammatory effects of OXY in macrophages with LPS stimulation [20]. Therefore, this study aimed to assess the anti-inflammatory effects of OXY on periodontal ligament cells induced by LPS. The null hypothesis was that lipopolysaccharide (LPS)-oriented inflammatory response in periodontal ligament (PDL) cells is not affected by OXY treatment.

## Methods

### Materials and cell culture

OXY (CAS No. 29700-22-9) was obtained from the National Institute for Korean Medicine Development (NIKOM; Gyeongsangbuk-do, Korea). LPS from *Porphyromonas gingivalis* was purchased from InvivoGen (San Diego, CA, USA). Human PDL cells (Lot No. 90I14-096) from a 28-year-old male were purchased from CEFO co., Ltd (Seoul, Korea) and cultured in Dulbecco’s Modified Eagle’s medium (DMEM; GibcoBRL, Life technologies, Grand Island, NY) with some supplements (10% fetal bovine serum and 1% streptomycin). The 4th and 5th generations cells cultured under 37 °C and 5% CO_2_ environment were used.

### Cell counting Kit-8 (CCK-8) test

After seeding PDL cells at a density of 5 × 10^3^, the cells were incubated for 24 h. OXY powder was diffused in dimethyl sulfoxide (Gibco BRL, Life technologies, Grand Island, NY), diluted in DMEM to concentrations of 0, 10, and 20 µg/mL, and then exposed to the cells for 72 h [21]. Then, the CCK-8 (Dojindo Molecular Technologies, Kumamoto, Japan) solution was treated at 20 µL per well and the plates were read on a plate spectrophotometer (AMR-100; Allsheng, Hangzhou, Zhejiang, China) at 450 nm wavelength. The experiments were performed in 5 replicates.

### Flow cytometry

PDL cells exposed to OXY solutions for 72 h were collected using trypsin-ethylenediaminetetraacetic acid (Giboco BRL, Life technologies, Grand Island, NY, USA) until they reached 1 × 10^5^ cells per well. The cells were placed in binding buffer (500 µL) and 5 µL of Annexin V-FITC (abcam; ab14085) and 7-AAD (abcam; ab228563) were added in the dark environment. Finally, the cells were evaluated by a flow cytometer (BD FACSVerse, BD Biosciences, NJ, USA) and analyzed with FlowJo software (Tree Star Inc., Ashland, OR, USA).

### Nitric oxide (NO) assay

PDL cells (*n* = 3 per group) seeded at a density of 2 × 10^5^ in 6 well plates were exposed to OXY solutions (0 and 10 µg/mL). After 1 h exposure to OXY solutions, LPS (10 µg/mL) was added and then incubated for 24 h. Optical density at 540 nm was evaluated after adding the buffers from a commercial kit (NO Plus Detection Kit; iNtRON Biotechnology, Inc., Seoul, Korea). NO production was quantified by NO standard curve according to the optical densities.

### Gelatin zymography

PDL cells were seeded at a density of 2 × 10^5^ in 6 well plates and treated with OXY and LPS in the same way as in the NO assay. The harvested supernatant was centrifuged and reacted with a zymography buffer kit containing 0.1% gelatin (CAT No. KZB010, LABISCOMA, Seoul, Korea). According to the manufacturer’s instructions, 1:10 dilution of buffers was used. The supernatants were diluted to zymogram sample buffer (CAT No. KZB020, LABISCOMA, Seoul, Korea) with a 1:1 ratio and loaded to be 10 µL. Then, electrophoresis of 125 V was performed for 1 h. The renaturing buffer was added for 30 min, developing buffer was applied, and incubated for 24 h. Coomassie Blue R-250 was added for 1 h. Image J (National Institutes of Health, Bethesda, MD, USA) software was used to evaluate the intensity of the bands. The percentage of the band area was calculated by comparing that of the control.

### Quantitative real-time polymerase chain reaction (qRT-PCR)

PDL cells were seeded at a density of 1 × 10^6^ in 6 well plates (*n* = 3 per group) and exposed to OXY solutions (0 and 10 µg/mL). The regimen for OXY and LPS was same as previously described. RNA was extracted using easy-BLUE and cDNA reverse transcription using Maxime RT PreMix (iNtRON Biotechnology). A Step One Plus real-time PCR machine (Applied Biosystems, Thermo Fisher Scientific, Inc., Waltham, MA, USA) was utilized for qPCR experiments using Power SYBR Green PCR Master Mix (Applied Biosystems). The levels of glyceraldehyde 3-phosphate dehydrogenase (GAPDH) were used to standardize the levels of cDNA using the 2^−ΔΔCt^ technique. The primer sequences utilized in this study are shown in Table 1 [22].

**Table 1:** Quantitative real-time polymerase chain reaction primer sequences

Gene	Accession No.	Primer (5’ − 3’)	Size (bp)	TM (°C)	GC (%)
*TNF-α*	NM_000594	Forward: AACATCCAACCTTCCCAAACGC Reverse: TGGTCTCCAGATTCCAGATGTCAGG	1678	60.9 62.6	50.00 52.00
*IL-6*	NM_000600	Forward: CGCCTTCGGTCCAGTTGCC Reverse: GCCAGTGCCTCTTTGCTGCTTT	1127	62.9 63.4	68.42 54.55
*IL-8*	NM_000584	Forward: CTCTTGGCAGCCTTCCTGATTTC Reverse: TTTTCCTTGGGGTCCAGACAGAG	1642	60.8 61.1	52.17 52.17
*GAPDH*	NM_002046	Forward: CATGAGAAGTATGACAACAGCCT Reverse: AGTCCTTCCACGATACCAAAGT	1285	57.5 58.1	43.48 45.45

TNF-α: tumor necrosis factor alpha, IL-6: interleukin 6, IL-8: interleukin 8, GAPDH: glyceraldehyde 3-phosphate dehydrogenase

### PDL cell migration/cell proliferation assay

PDL cells were transferred on culture dishes with SPLScar Block (SPL Life Sciences, Gyeonggido, Korea). After SPLScar™ Block was gently removed using sterile forceps, the cells (*n* = 8 per group) were treated in the same way as in the NO assay. The cell images were taken for 48 h using a JuLI microscope (NanoEnTek, Seoul, Korea). The shortest distance between the margins were measured. The open wound area (%) was obtained as follows: open wound area (%): gap length at evaluated time / gap length at 0 h × 100%.

### Statistical analysis

Data was shown as mean ± standard deviation. IBM SPSS Statistics v. 20.0 (IBM Corp., Armonk, NY, USA) was used for the statistical analysis, and the one-way ANOVA test and Tukey’s HSD post-hoc test were used. *P*-values < 0.05 indicated statistical significance.

## Results

**Fig. 1:**
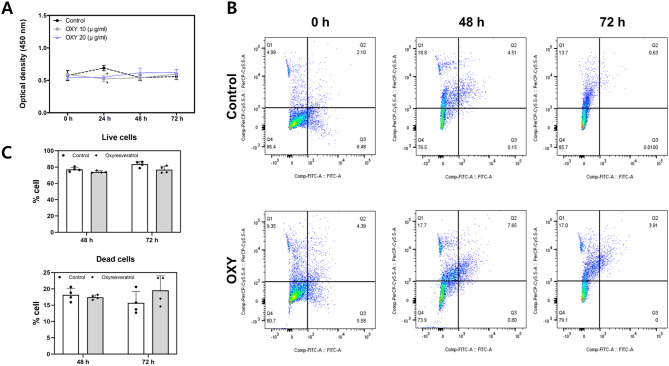
PDL cell viabilities treated by OXY. (**A**) CCK-8 test. The PDL viabilities were decreased in OXY-treated groups at 24 h (^*^p < 0.05) and then not affected by OXY after 48 h. (**B**) Annexin V-FITC/7-AAD apoptosis staining representative dot plots by flow cytometry. Live cells are shown as dots in the Q4 section and dead cells in the Q2 section. (**C**) Bar graphs of live and dead cells of OXY (0 and 10 µg/mL). ^*^p < 0.05 vs. control. Control: No treatment, OXY: Oxyresveratrol

We confirmed that cell viability was not affected by OXY treatment. As measured using CCK-8, there was no significant effect on cell viability at any concentration when exposed to OXY for 72 h (Fig. 1A). Confirming that OXY had no effect on cell viability, the flow cytometry experiment, which was similar to the CCK-8 results, also revealed that OXY 10 µg/mL did not significantly affect the percentage of live cells when compared to the control (Fig. 1B and C).

**Fig. 2:**
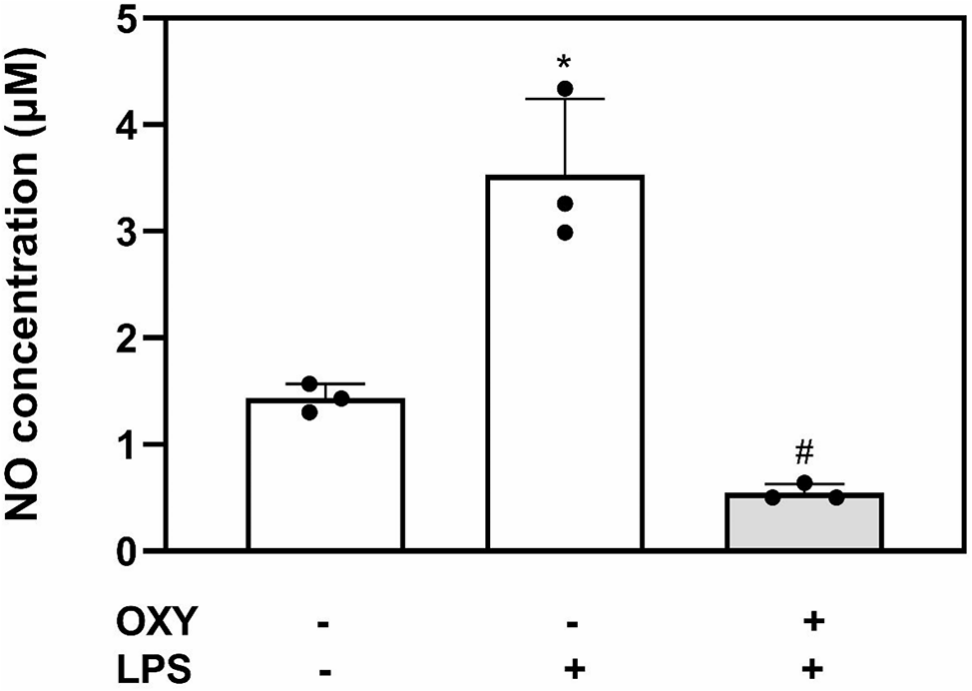
Nitric oxide (NO) assay. OXY (10 µg/mL) treatment significantly decreased the amount of NO produced in response to LPS stimulation (^*^p < 0.05 vs. Control, ^#^*p* < 0.05 vs. LPS). OXY: Oxyresveratrol, LPS: lipopolysaccharide

Regarding NO production, After 24 h of LPS stimulation, NO production was significantly increased (Fig. 2). OXY (10 µg/mL) treatment significantly decreased the NO production under 24 h of LPS stimulation (*p* < 0.05). However, significant differences in NO production were not observed between two groups.

**Fig. 3:**
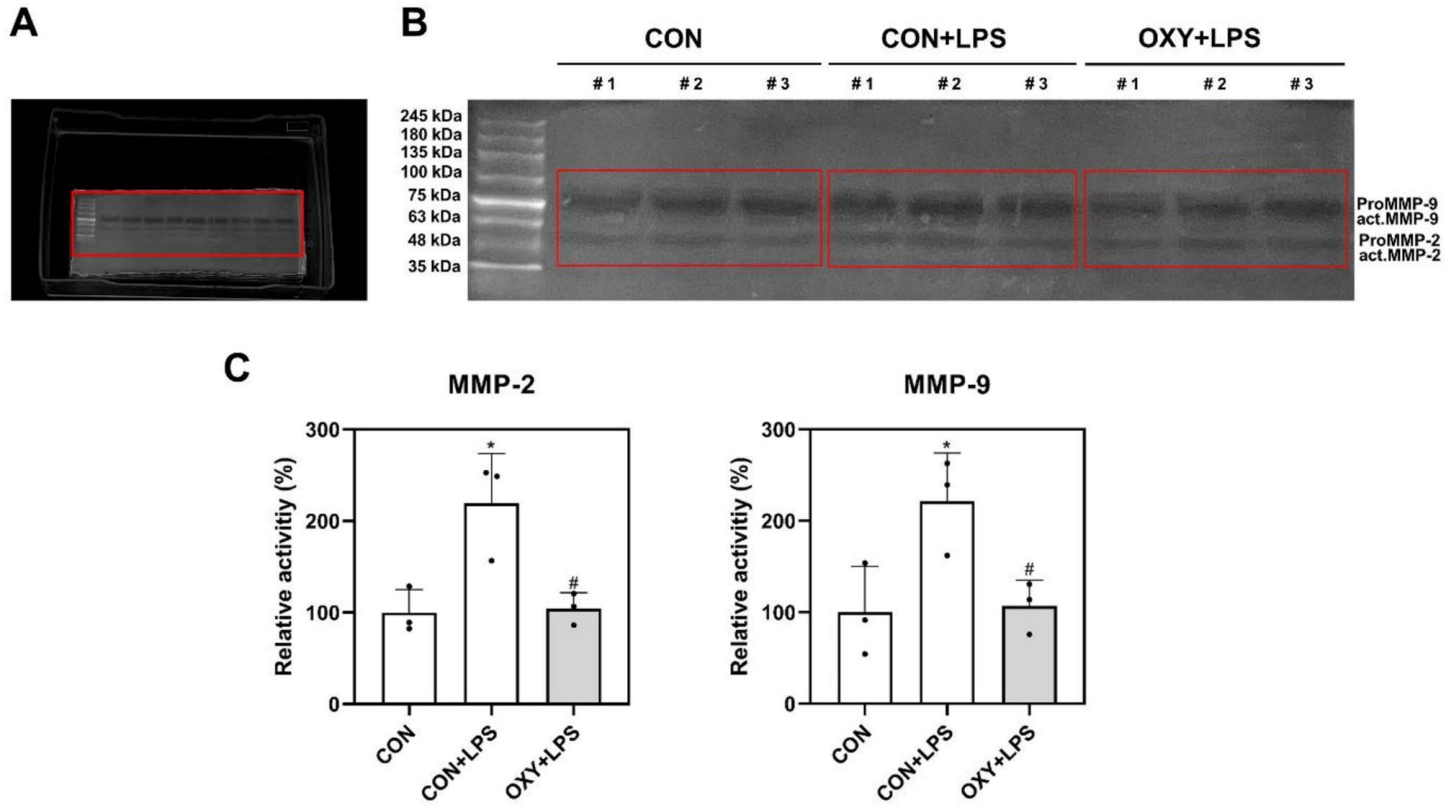
Gel zymography for matrix metalloproteinases-2 (MMP-2) and matrix metalloproteinase-9 activity (MMP-9). (**a**) Full length MMP zymography without cutting gel/blot. (**b**) Representative images of MMP zymography. MMP activity presents in the form of bands. (**c**) Relative activity (%) of MMP-2 and MMP-9. ^*^p < 0.05 vs. Control, ^#^*p* < 0.05 vs. LPS. CON: control, OXY: Oxyresveratrol, LPS: lipopolysaccharide

**Fig. 4:**
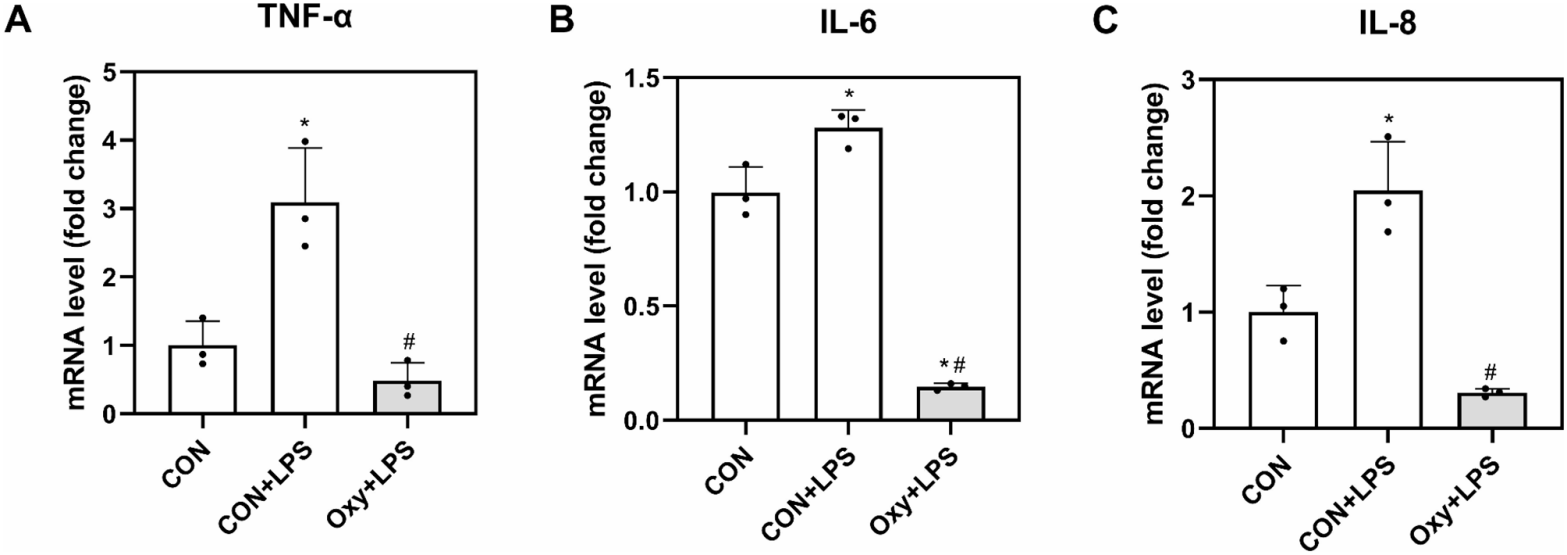
Expression of pro-inflammatory cytokine markers. (**A**) TNF-α, (**B**) IL-6, (**C**) IL-8. ^*^p < 0.05 vs. Control, ^#^*p* < 0.05 vs. LPS. CON: control, OXY: Oxyresveratrol, LPS: lipopolysaccharide

Figure 3 shows the effect of OXY on MMP-2 and MMP-9 expression. MMP-2 and MMP-9 activity was increased after LPS stimulation. LPS-induced MMP-2 and MMP-9 activation was significantly inhibited by OXY (10 µg/mL) treatment. TNF-α, IL-6, and IL-8 expression by OXY treatment is shown in Fig. 4. Pro-inflammatory cytokine expression levels were significantly increased following LPS treatment (*p* < 0.05). However, the level of inflammatory expression was significantly inhibited by OXY (10 µg/mL) treatment (*p* < 0.05).

**Fig. 5:**
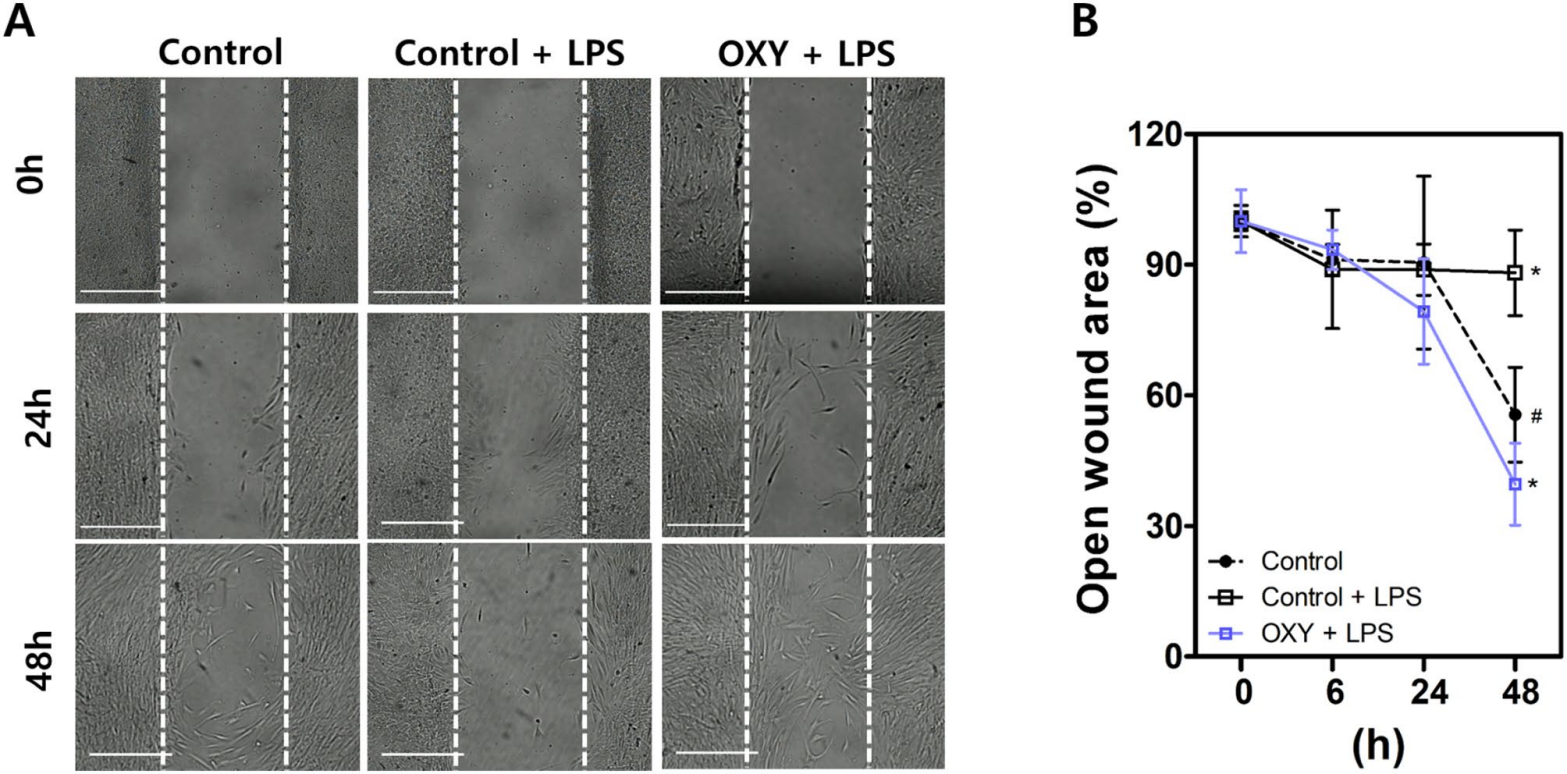
Cell migration/cell proliferation assay. (**A**) Representative images, (**B**) Results of open wound area (%). Scale bar = 500 μm. ^*^p < 0.05 vs. Control, ^#^*p* < 0.05 vs. OXY + LPS. OXY: Oxyresveratrol, LPS: lipopolysaccharide

To assess effects of OXY on PDL cell migration and proliferation under periodontal inflammation, cell migration/cell proliferation assay was performed (Fig. 5). There were no significant differences in open wound area among the groups for 24 h. At 48 h, open wound area was significantly lower in OXY (10 µg/mL) treatment group (39.57 ± 8.79%) compared with the control (55.56 ± 10.20%) and control + LPS groups (88.12 ± 9.19%) (*p* < 0.05).

## Discussion

We assessed the in vitro effect of OXY on human PDL cells stimulated by LPS. This study showed that OXY significantly inhibited NO production and pro-inflammatory cytokines expression under LPS stimulation. Moreover, OXY promoted PDL cell migration/proliferation under LPS stimulation. Thus, the null hypothesis was rejected.

This study found that NO production by LPS stimulation was inhibited by OXY treatment. This finding is corresponded to previous in vitro studies [23, 24]. A recent study found that OXY treatment up to 40 µmol/L significantly reduced LPS-stimulated NO production and this inhibition was to be dose dependent of OXY [23]. NO is a key signaling molecule involved in inflammation [25]. During the inflammation phase, pro-inflammatory cytokine expression leads to NO production. Thus, inhibition of NO production may be related to a reduction in the inflammatory response associated with periodontitis.

In this study, OXY significantly reduced LPS-stimulated expression of MMP-2 and MMP-9. This finding is corresponded to the results of previous studies on human melanoma cell [26]. MMPs mainly participate in the pathways associated with periodontal tissue destruction [27]. Periodontal pathogen secretes specific proteases that cause pro-MMP-2, which is secreted by the periodontal ligament cells in a form that is inactive, to become active and these proteases cause periodontal destruction [28]. Because of this, the MMP-2 is involved in the inflammatory process, collagen formation, and alveolar bone regeneration [29]. MMP-9 is produced during the degradation of connective tissue in inflammatory conditions, such as periodontal disease, and is essential component of the osteoclast resorption process [30]. And its elevation is related to inflammatory process, particularly severe periodontal tissue destruction [31]. Moreover, a previous study found that a close link between elevation of MMP-2 and MMP-9 activity and inflamed periodontal tissue and tissue damage [32]. Thus, the inhibition of OXY treatment in MMP-2 and MMP-9 activity might be beneficial in controlling periodontal inflammation.

OXY significantly reduced LPS-induced expression levels of pro-inflammatory cytokines. This finding is consistent with previous studies. As the relationship between TNF-α expression and periodontal disease has been well-documented [33], it may be used as an indicator for treating and responding to periodontitis. A previous study confirmed an improvement in colitis by OXY treatment in a mouse model and downregulation of TNF-α by OXY treatment [34]. Another study with a mouse model of gastric ulcer found that 50 mg/kg of OXY treatment significantly reduced the production of TNF-α [35]. Moreover, IL-6 is associated with alveolar bone resorption [29], and IL-8 with its effect on neutrophils, investigation of IL-6 and IL-8 expression in patients with periodontal disease may be used as an indicator of treatment response [36]. A recent study found that a significant reduction in IL-6 and IL-8 expression in keratinocyte by OXY treatment [37]. Based on the previous finding, it might be hypothesized that OXY may regulate MAPK and NF-κB pathways, thereby reducing the expression of these cytokines [38]. Previous studies have shown that resveratrol, an OXY-like compound, can alleviate periodontitis through inhibition of the NF-κB pathway, which is essential to produce LPS-induced inflammatory cytokines and MMPs [39]. Also, polyphenols showed therapeutic effects on periodontal disease by inhibiting LPS-induced inflammatory cytokines (IL-1β, IL-6, IL-8) in PDL cells [40]. These results are very similar to our findings and suggest that OXY can be applied as a therapeutic agent for periodontal disease.

Regarding PDL cell migration/proliferation, LPS inhibited cell migration/proliferation and OXY promoted cell migration/proliferation in LPS-induced inflammatory environment. These finding indicate that OXY may prevent periodontitis by creating an environment that facilitates periodontal regeneration under periodontal inflammation. Because PDL cells are the primary supplier of progenitor cells, they are critical for periodontal regeneration [41, 42]. As a result, it is vital to recruitment and proliferation of PDL cells during periodontal tissue destruction [43, 44].

However, this study has limitations. First, effects of OXY were only evaluated in cellular level. Thus, future studies with animal models will be necessary to assess the effects of OXY in periodontal inflammation. Second, identification of signaling pathways related to OXY treatment was not considered. Further studies evaluating in protein levels will also be necessary.

## Conclusions

In conclusion, this study evaluated the effects of OXY in human PDL cells under periodontal inflammation and found that OXY promoted cell migration/proliferation and inhibited periodontal inflammatory responses. This indicates that OXY may be promising for the prevention of periodontitis since it regulates inflammatory responses and promotes the periodontal regeneration. However, the translation to clinical practice remains uncertain without further research due to the nature of this in vitro study.

## Electronic supplementary material

Below is the link to the electronic supplementary material.

Supplementary Material 1

## Data Availability

All data generated or analyzed from this study are included in this published article.
