# Supplementary material for: Effect of oxyresveratrol under in vitro lipopolysaccharide-induced periodontitis environment
Source: BMC Oral Health. 2024 Nov 15;24:1382. doi: 10.1186/s12903-024-05128-2 (PMC11566898; doi:10.1186/s12903-024-05128-2)
Supplement: Supplementary file 1 — Supplementary Material 1 [file 12903_2024_5128_MOESM1_ESM.docx]

**Additional file 1**


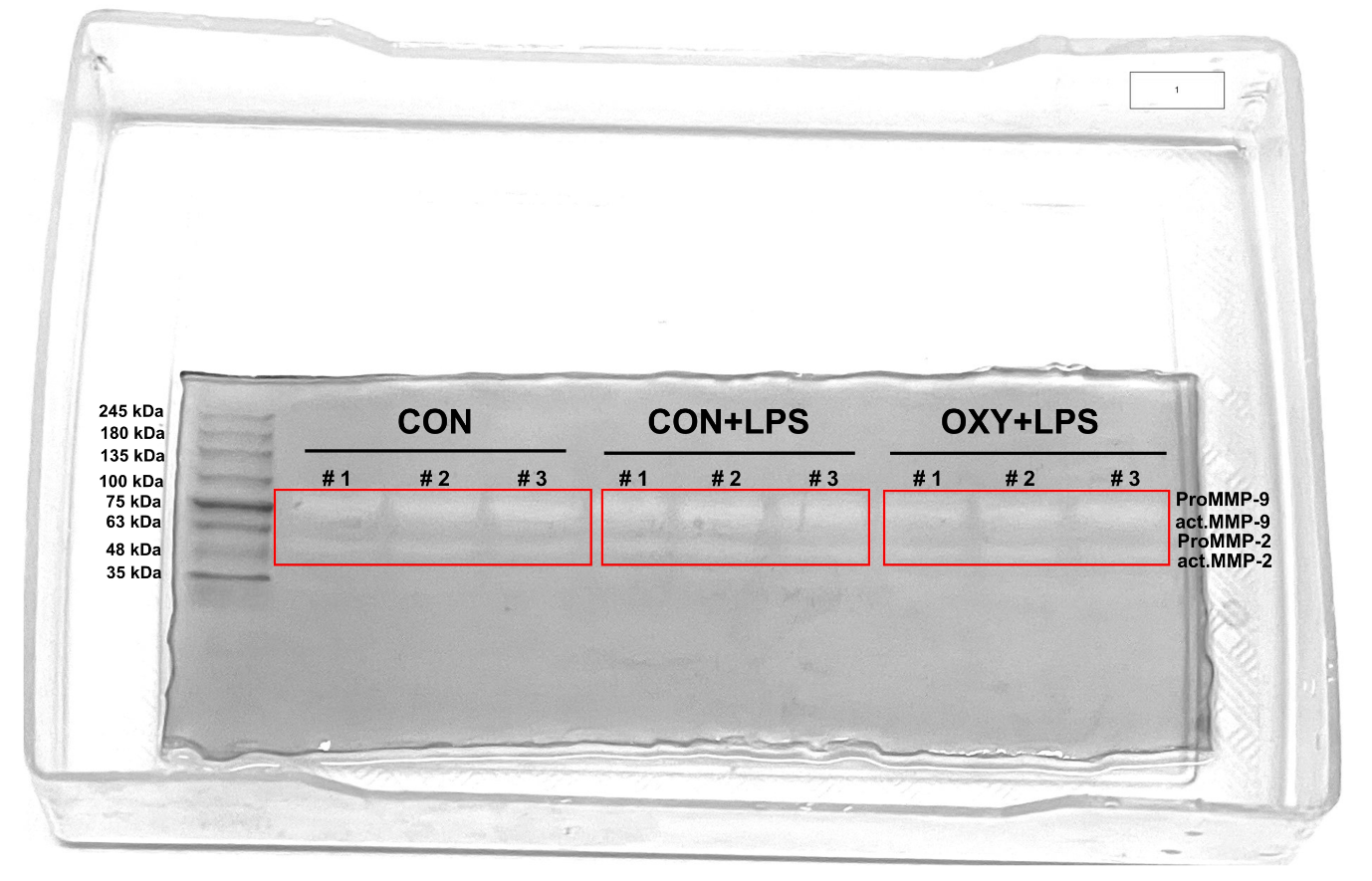


**Supplementary figure 1.** Full-length MMP zymography without cutting gels/blots for matrix metalloproteinase-2 (MMP-2) and matrix metalloproteinase-9 activity (MMP-9). CON: control, OXY: Oxyresveratrol, LPS: lipopolysaccharide.


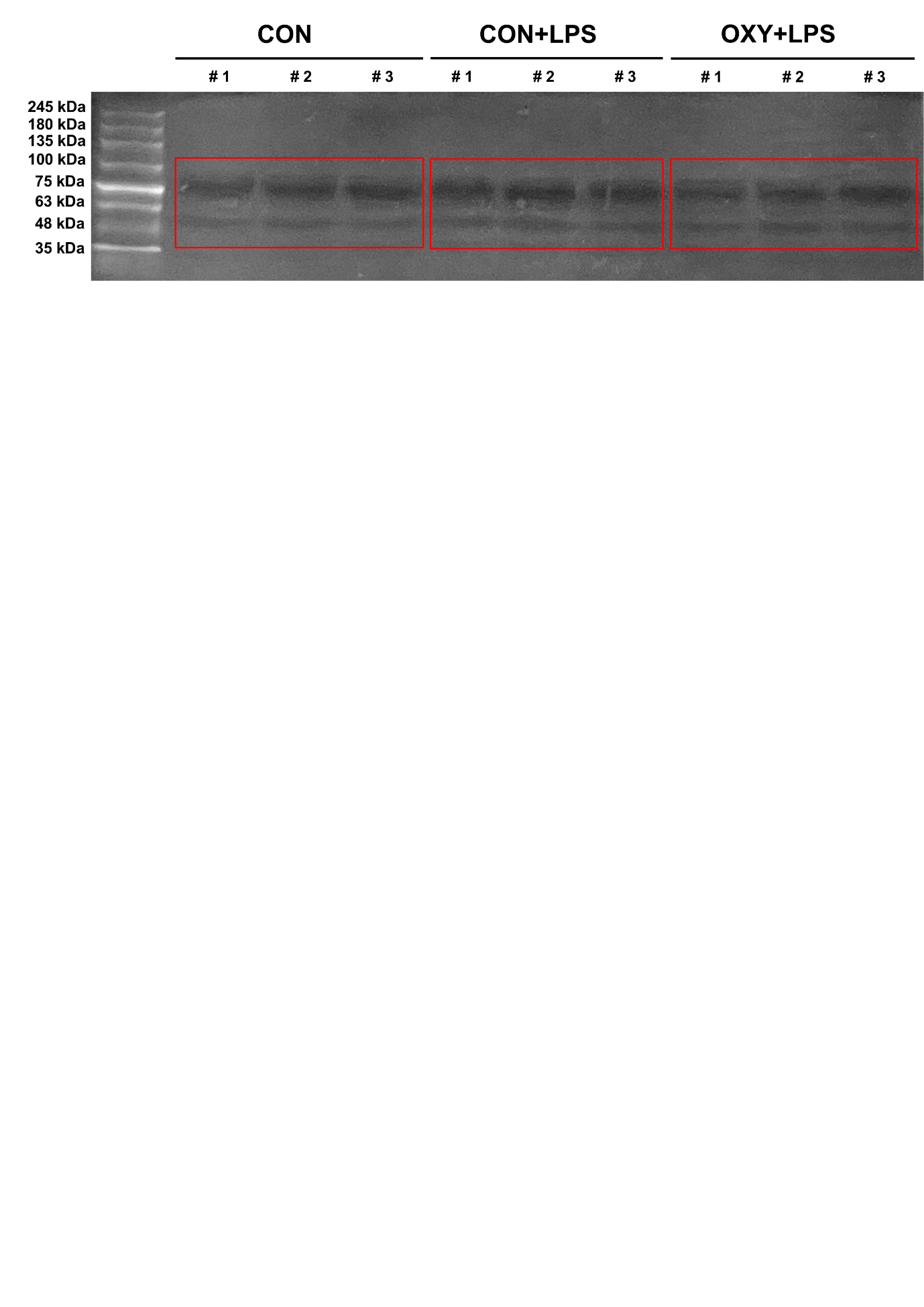


**Supplementary figure 2.** Representative images of MMP zymography. MMP activity presents in the form of bands. CON: control, OXY: Oxyresveratrol, LPS: lipopolysaccharide.


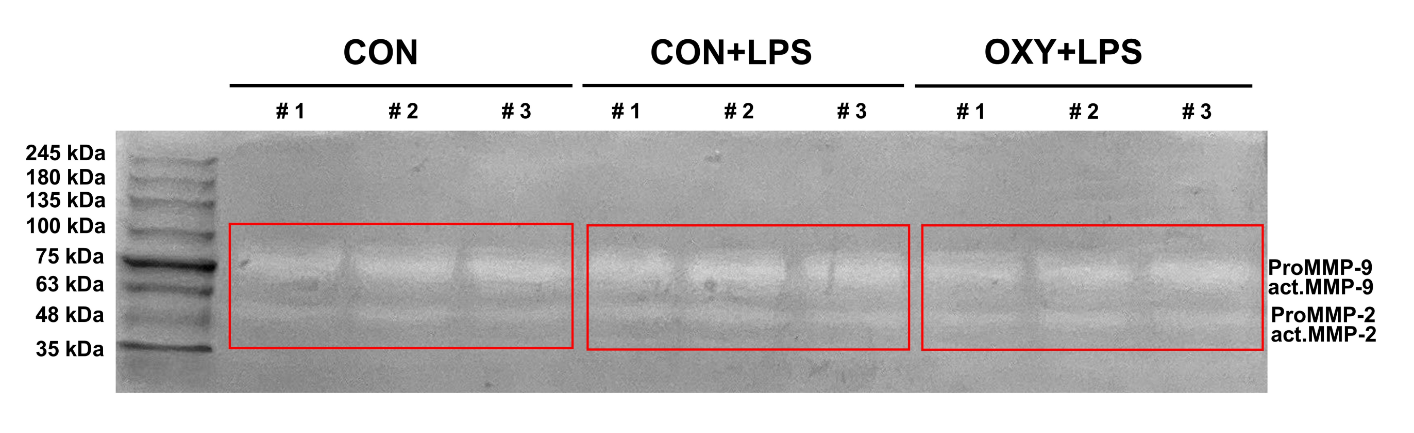


**Supplementary figure 3.** Representative MMP zymography image with different exposures CON: control, OXY: Oxyresveratrol, LPS: lipopolysaccharide.
